# 1982. GLP-1 Receptor Agonists Promote Weight Loss Among People with HIV

**DOI:** 10.1093/ofid/ofad500.109

**Published:** 2023-11-27

**Authors:** Quynh P Nguyen, Darcy Wooten, Kye Duren, Manuel Moreno, Katherine Promer, Matthew Hien Tan, Michael E Tang, Amutha V Rajagopal, Lucas Hill, Jeffrey Yin, Will Toperoff

**Affiliations:** University of California, San Diego, San Diego, CA; University of California, San Diego, San Diego, CA; UCSD, La Jolla, California; UC San Diego, San Diego, California; UC San Diego Health, San Diego, California; UC San Diego, San Diego, California; University of California, San Diego, San Diego, CA; UC San Diego, San Diego, California; University of California, San Diego, San Diego, CA; University of California, San Diego, San Diego, CA; University of California, San Diego, San Diego, CA

## Abstract

**Background:**

Weight gain and associated metabolic complications are increasingly prevalent among people with HIV (PWH), especially those initiating second-generation integrase strand transfer inhibitors (INSTIs). Glucagon-like peptide-1 receptor agonists (GLP-1 RAs) are incretin-based therapies for diabetes that have been shown to result in substantial weight loss; however, studies of their efficacy in PWH are limited. We aimed to describe the prescribing practices and clinical outcomes of GLP-1 RA use among PWH.

**Methods:**

We conducted a retrospective cohort study among PWH who were prescribed GLP-1 RAs at University of California, San Diego between 2/1/2021and 2/1/2023. Patients who were prescribed but never took GLP-1 RAs or those for whom weight data were not available after GLP-1 RA initiation were excluded. We collected baseline clinical data and calculated changes in weight, body mass index (BMI), and hemoglobin A1C (A1C) before and during receipt of GLP-1 RA. We conducted logistic regression to identify variables associated with more than 5% of total body weight loss.

**Results:**

227 patients met our inclusion criteria. Baseline characteristics are shown in Table 1. Ninety-nine patients (43%) were prescribed GLP-1 RAs for weight management alone without concurrent diabetes. Patients had received on average 15.2 months of GLP-1 RA therapy, with 92 (40.5%) achieving the maximum GLP-1 RA dose. On average, GLP-1 RA therapy resulted in a loss of 12 pounds, decrease in BMI by 1.8, and decrease in A1C by 0.5 among all patients and by 1.2 among patients with an A1C > 6.5 at baseline. In the multivariable analysis, higher baseline BMI [OR 1.07 (1.02-1.3)] and longer treatment duration of GLP-1 RA therapy [OR 1.04 (1.01-1.07)] were significantly more likely to be associated with >5% weight loss, whereas receipt of dulaglutide significantly decreased the likelihood of >5% weight loss compared to other GLP-1 RAs [OR 0.33 (0.17-0.66)]. Age, sex assigned at birth, race, ethnicity, ART regimen, baseline CD4 cell count, HIV viral load, and presence of diabetes were not predictive of weight change.

**Table 1.**
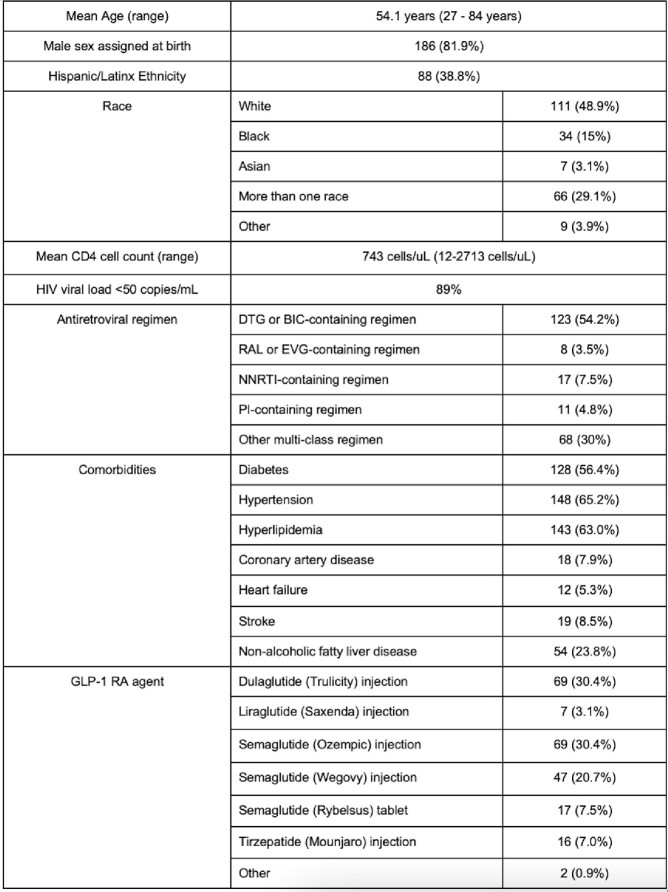
Baseline Patient Characteristics (N = 227)

**Table 2.**
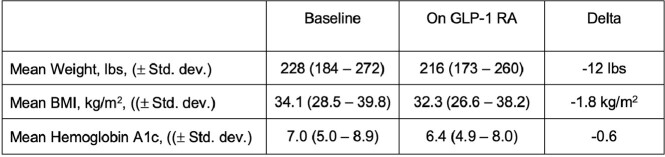
Mean weight, BMI, and A1C before and during GLP-1 RA therapy for PWH

**Conclusion:**

Use of GLP-1 RAs led to improvements in weight, BMI, and hemoglobin A1C among PWH and offers an additional strategy to address weight gain and related metabolic complications.

**Disclosures:**

**All Authors**: No reported disclosures

